# A bibliometric analysis of abdominal aortic aneurysm (2014–2024)

**DOI:** 10.3389/fcvm.2024.1436600

**Published:** 2024-12-13

**Authors:** Shao-Jia Liu, Xin-Qing Yang, Hong-Qiao Lu, Kun-Chi Zhang, Yong-Jiang Tang, Yu Xu

**Affiliations:** ^1^ Panzhihua Central Hospital, Panzhihua, Sichuan, China; ^2^ The Affiliated Hospital of Kunming University of Science and Technology, Kunming, China

**Keywords:** abdominal aortic aneurysm, bibliometric analysis, VOSviewer, CiteSpace, R package

## Abstract

**Background:**

Abdominal aortic aneurysm (AAA) is a localized bulge of the abdominal aorta, which mainly manifests as a pulsatile mass in the abdomen. Once an abdominal aortic aneurysm ruptures, the patient's life is seriously endangered. Surgery is the preferred treatment for abdominal aortic aneurysm. At present, there has been no comprehensive review of the current status of abdominal aortic aneurysm research. Therefore, this study aimed to identify global trends in abdominal aortic aneurysm research over the last 10 years through bibliometric analysis and to inform clinical practice, research funding allocation, and decision-making.

**Methods:**

We downloaded research articles and reviews on abdominal aortic aneurysm from 1 January 2014, to 1 March 2024, from the Web of Science core collection. CiteSpace (version 6.2.1), RStudio and VOSviewer (version 1.6.18) were used for visual analysis of regional distribution, institutions, authors, keywords and other information.

**Results:**

The number of documents on abdominal aortic aneurysm research increased continuously and has stabilized in recent years. A total of 9,905 publications from 67 countries were published from 1 January 2014, to 1 March 2024. A total of 2,142 (29.52%) studies were from the United States, 1,293 (13.05%) were from China, and 919 (9.28%) were from the United Kingdom. A total of 205 studies were conducted at Stanford University, 172 were conducted at Harvard Medical School, and 165 were conducted at the Mayo Clinic. The top three coauthorship authors were Schermerhorn, Marc L (114); Golledge, Jonathan (102); and De Vries, Jean Paul P.M. (74). The most cocited reference was Chaikof EL, 2018, J Vasc Surg, v67, p. 2; the most cocited journal was the Journal of Vascular Surgery; and the most cocited author was Lederle, FA. “Abdominal aortic aneurysm” was the most frequently used author keyword (2,492). Twenty-five references with strong citation bursts were identified by “CiteSpace”. “Artificial intelligence”, “clinical outcomes” and “bridging stent” were the primary keywords of emerging research hotspots.

**Conclusion:**

This is the first bibliometric study to comprehensively summarize the research trends in abdominal aortic aneurysm research. This information can help us to identify the current research hotspots and directions. This study will provide extensive help for future research.

## 1 Introduction

Abdominal aortic aneurysm (AAA) is a localized expansion of the abdominal aorta, with a diameter greater than 3 mm. The main sign is a palpable fluctuating mass in the abdomen, which may be asymptomatic, and is usually detected by physical examination (1, 2). Relevant statistics show that the incidence of abdominal aortic aneurysm is closely related to hypertension (3), smoking status (4), male sex (5) and other factors (6). Epidemiological studies have shown an increasing incidence of abdominal aortic aneurysm in men over 60 years of age (7). In the process of aneurysm formation, inflammatory reactions (8), loss of elastic fibers (9) and degradation of collagen (10) are the pathological mechanisms underlying the occurrence and development of abdominal aortic aneurysm. Abdominal aortic aneurysm rupture is the most common cause of death (11). Conservative treatment of abdominal aortic aneurysm is limited, and the main plan involves controlling blood pressure and heart rate (12). Surgical treatment, including open surgery and endovascular interventional surgery (13), is the most effective method for reducing the risk of descending abdominal aortic aneurysm. Endovascular interventional surgery has become the first choice for the treatment of abdominal aortic aneurysm because it involves less trauma, less bleeding, and fewer complications (14).

With improvements in clinicians' awareness of various diseases, especially complex diseases, an increasing number of high-quality studies on abdominal aortic aneurysm have been published (15, 16). For clinicians and researchers, staying well-informed about the most recent research and current hot topics related to abdominal aortic aneurysm can not only contribute to scientific research but also have a practical effect on clinical practice.

At present, there is no quantitative analysis of the relevant literature on abdominal aortic aneurysm. A real-time understanding of the status quo of abdominal aortic aneurysm research can help researchers develop specific ideas and gain greater value, and research on abdominal aortic aneurysm can be further refined and in depth. In view of the research hotspots in various fields, bibliometrics, which can be used to more clearly grasp the research hotspots and frontiers of the target field and provide readers with clearer ideas, plays an irreplaceable role (17, 18).

In this study, literature on abdominal aortic aneurysm published from 1 January 2014, to 1 March 2024, was retrieved from the Web of Science core collection database, and bibliometric statistical methods were used to understand the current research hotspots and directions of abdominal aortic aneurysm research to provide statistical support for future research in this field.

## 2 Materials and methods

### 2.1 Data collection and collation

**Figure 1:**
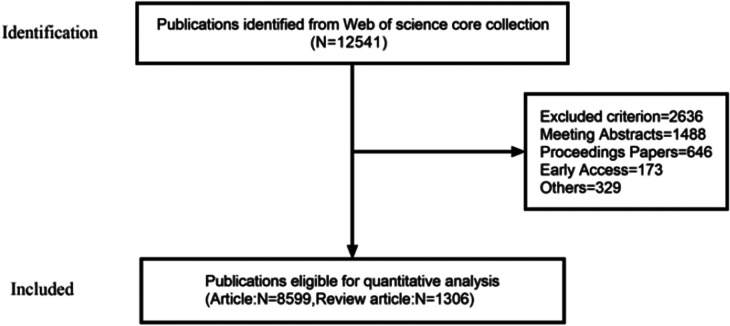
Publications screening flowchart.

We searched the Web of Science Core Collection for articles on abdominal aortic aneurysms from 1 January 2014, to 1 March 2024. The search formula was ([TS = (Abdominal aortic aneurysm)] OR TS = (Abdominal Aortic Aneurysms)) OR TS = (Abdominal Aorta Aneurysm)) OR TS = (Abdominal Aorta Aneurysms)) AND LA = (English). The time frame was from 1 January 2014, to 1 March 2024, and the type of documents was set to “articles” and “review” (Figure 1).

### 2.2 Data analysis

In this study, VOSviewer software was used for bibliometric analysis, including author, organization, country, journal and cocited journal, author and cocited author, and keyword co-occurrence analyses (19). CiteSpace software was mainly used to analyze references with citation bursts (20). The R package was mainly used for related subjective analysis, construction of a global distribution network and trend hotspot analysis of abdominal aortic aneurysm research (21). Excel software was used to describe the trend in publication volume. GraphPad Prism 8 was used for plot production and collation.

## 3 Results

### 3.1 Publication trends

**Figure 2:**
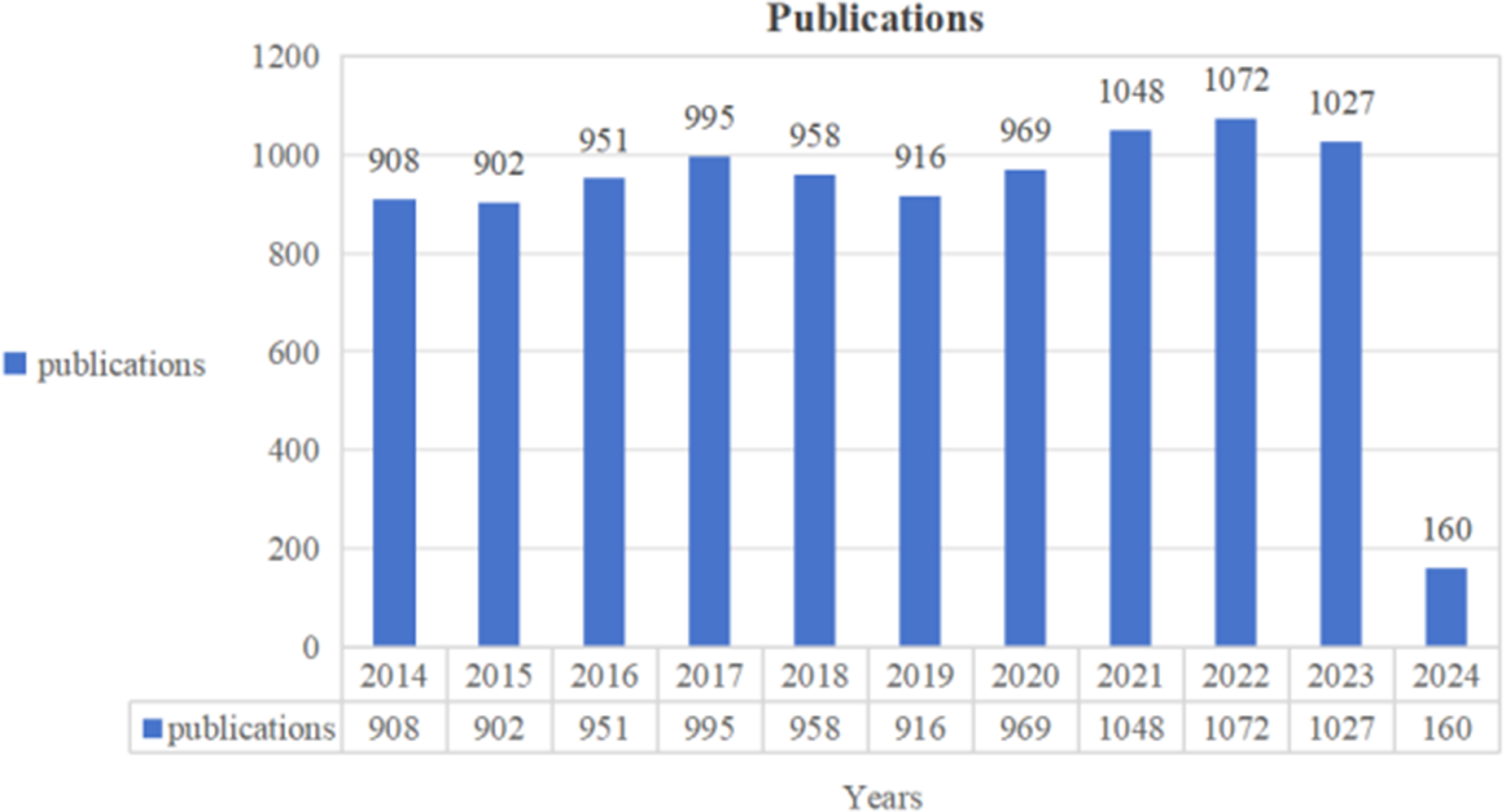
Annual output of research of abdominal aortic aneurysm.

We searched the literature on abdominal aortic aneurysm from the Web of Science Core Collection database and identified 9,905 articles and reviews published between 1 January 2014, and 1 March 2024. Overall, the number of articles on abdominal aortic aneurysm remained stable and slightly increased throughout the years (Figure 2). By the end of 2,023, the average annual number of publications was 9,746. Since 2014, the annual number of publications has been stable at more than 900. In the first two months of 2024, 160 articles on abdominal aortic aneurysm were published, indicating that abdominal aortic aneurysm research has been supported by many researchers.

### 3.2 Analysis of authors, institutions and countries

**Figure 3:**
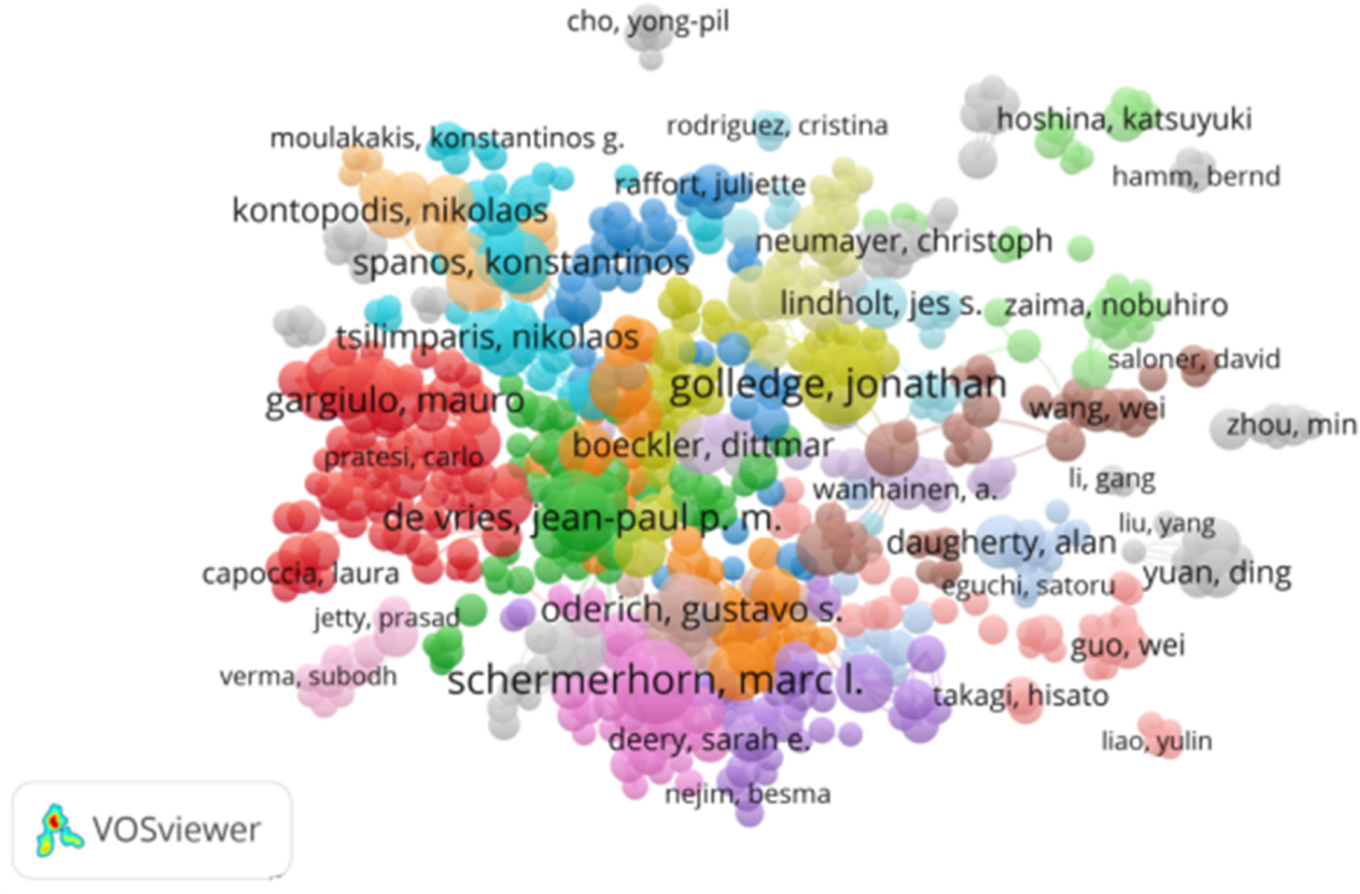
The visualization of collaborative networks between authors of abdominal aorticaneurysm-related studies.

A total of 36,397 authors with 9,905 publications in this field were identified. The 10 most productive authors are listed in Table 1. “Schermerhorn, Marc L” was the most prolific author (114 publications), followed by Golledge, Jonathan (102 publications) and De Vries, Jean-Paul PM. (74 publications). The cocited author network was generated as shown in Figure 3.

**Table 1:** Top 10 productive authors in the field of abdominal aortic aneurysm.

Rank	Author	Counts
1	schermerhorn, marc l.	114
2	golledge, jonathan	102
3	de vries, jean-paul p. m.	74
4	oderich, gustavo s.	70
5	wanhainen, anders	65
6	gargiulo, mauro	64
7	verhagen, hence j. m.	63
8	spanos, konstantinos	63
9	reijnen, michel m. p. j.	63
10	goodney, philip p.	56

**Figure 4:**
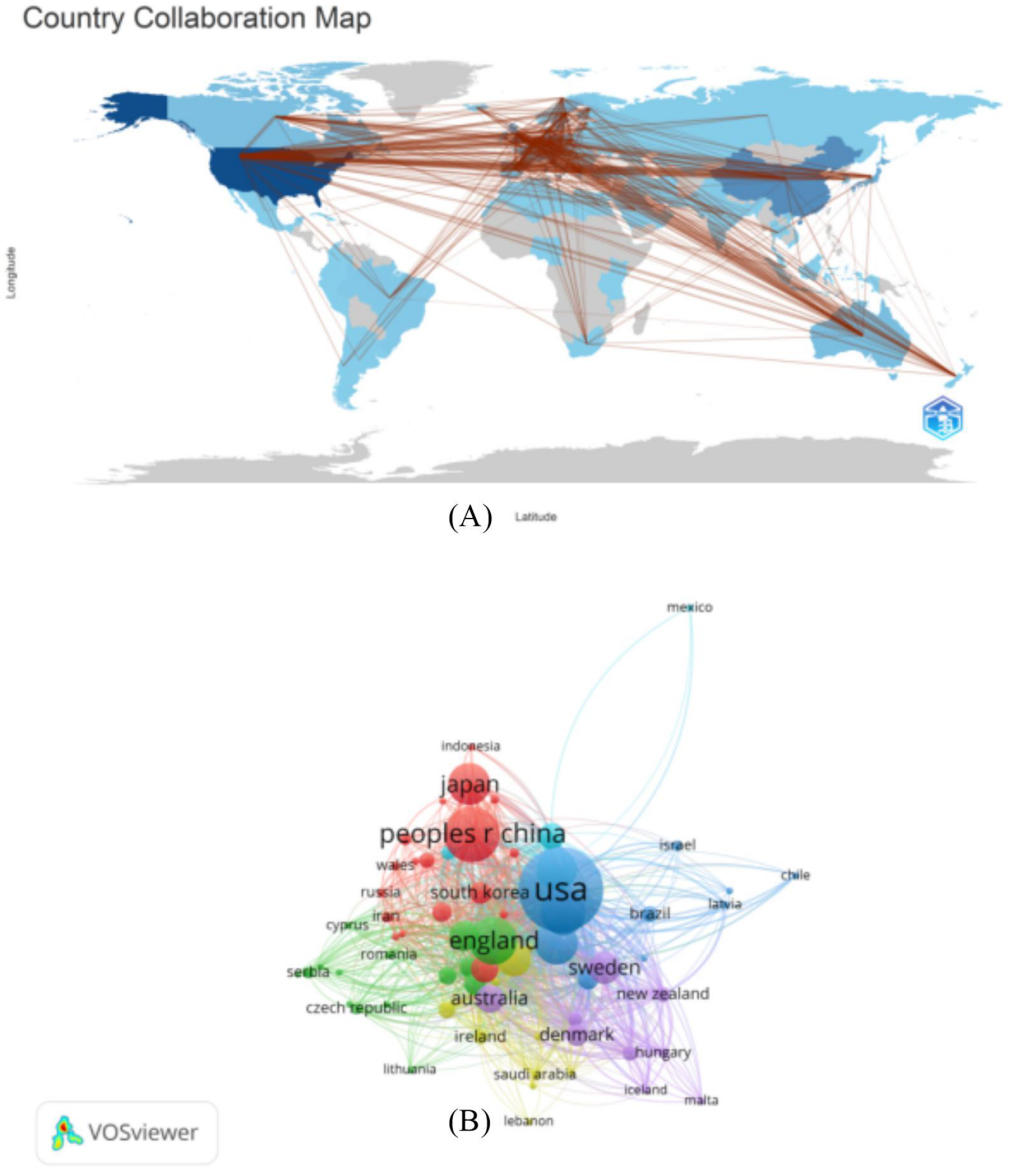
The geographic distribution of countries **(A)** and visualization **(B)** of studies on abdominal aortic aneurysms.

From 1 January 2014, to 1 March 2024, research related to abdominal aortic aneurysm was published by authors in 98 countries. The top ten countries were the United States, China, the United Kingdom, Germany, Italy, Japan, the Netherlands, France, Sweden and Greece, with a collective total of 9,373 articles published (94.63%). China ranked second with 1,293 articles, and the United Kingdom ranked third with 919 articles. China and the United States accounted for more than 40% (42.57%) of the publications. Moreover, we constructed a collaborative network based on the number of publications and relationships in each country (Table 2 and Figures 4A,B).

**Table 2:** Top 10 productive institutions and countries in the field of abdominal aortic aneurysm.

Rank	Institution	Counts	Country	Counts
1	Stanford Univ	205	USA	2,924
2	Harvard Med Sch	172	Peoples R China	1,293
3	Mayo Clin	165	England	919
4	Uppsala Univ	142	Germany	824
5	Karolinska Inst	134	Italy	754
6	Univ Michigan	118	Japan	723
7	James Cook Univ	112	Netherlands	675
8	Beth Israel Deaconess Med Ctr	105	France	466
9	Dartmouth Hitchcock Med Ctr	104	Sweden	434
10	Univ Pittsburgh	101	Greece	361

**Figure 5:**
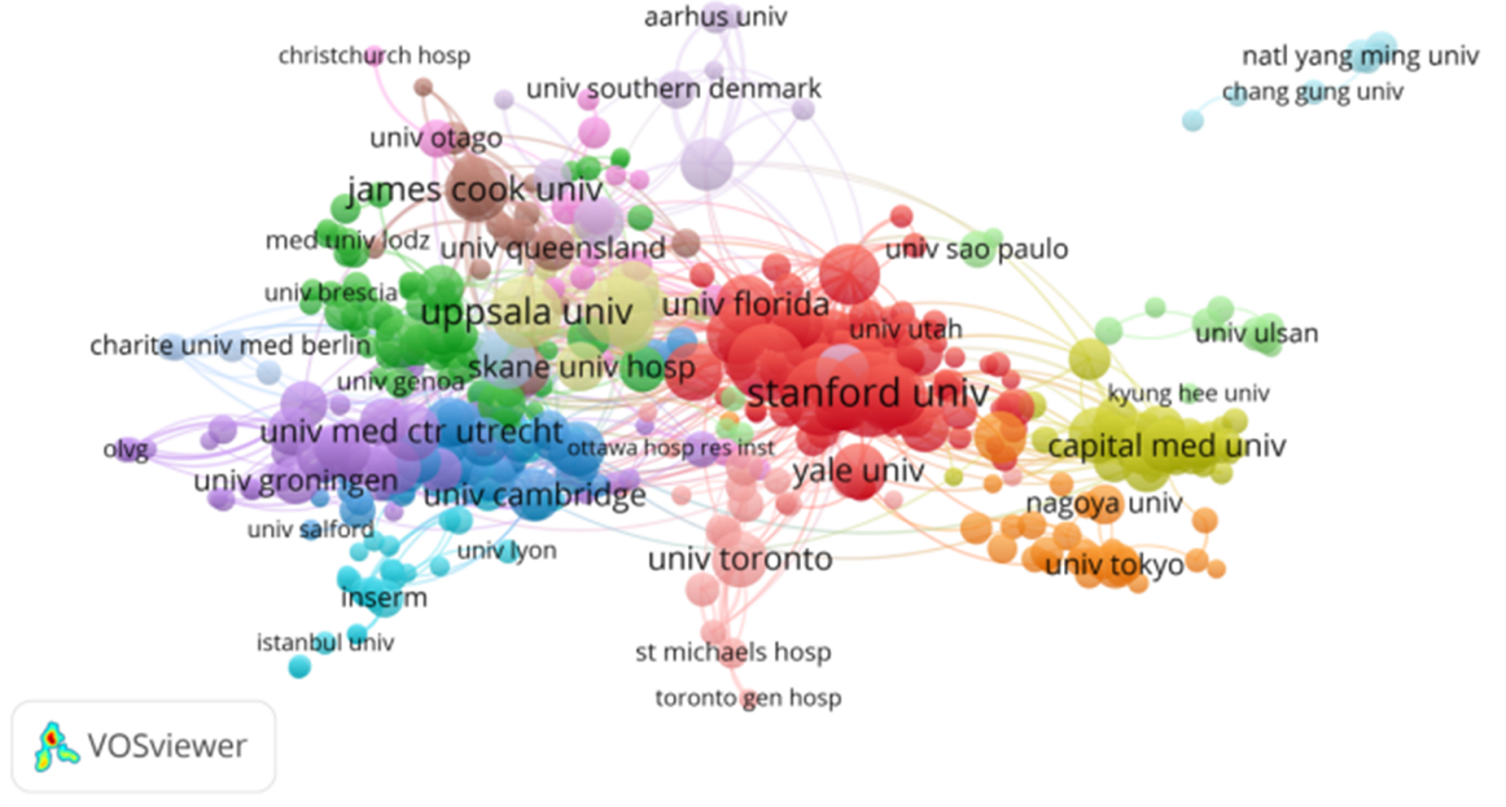
The visualization of institutions on research ofthe abdominal aortic aneurysm.

Researchers at 7,148 institutions published research on abdominal aortic aneurysm. The top 10 institutions were Stanford Univ, Harvard Med Sch, Mayo Clin, Uppsala Univ, Karolinska Inst, Univ Michigan, James Cook Univ., Beth Israel Deaconess Med Ctr, Dartmouth Hitchcock Med Ctr, and Univ. Pittsburgh. Stanford University had the largest number of published articles (205), followed by Harvard Med Sch (172) and the Mayo Clin (165). Subsequently, we constructed a collaborative network based on the number of publications and relationships of each institution (Table 2 and Figure 5). It is not difficult to see from the figure that the top institutions have close cooperation with multiple universities and have published more papers. At the same time, there are a few institutions that have established cooperative relations with only a few other institutions (Kaohsiung Med Univ).

### 3.3 Co-occurrence analysis of authors' keywords

**Figure 6:**
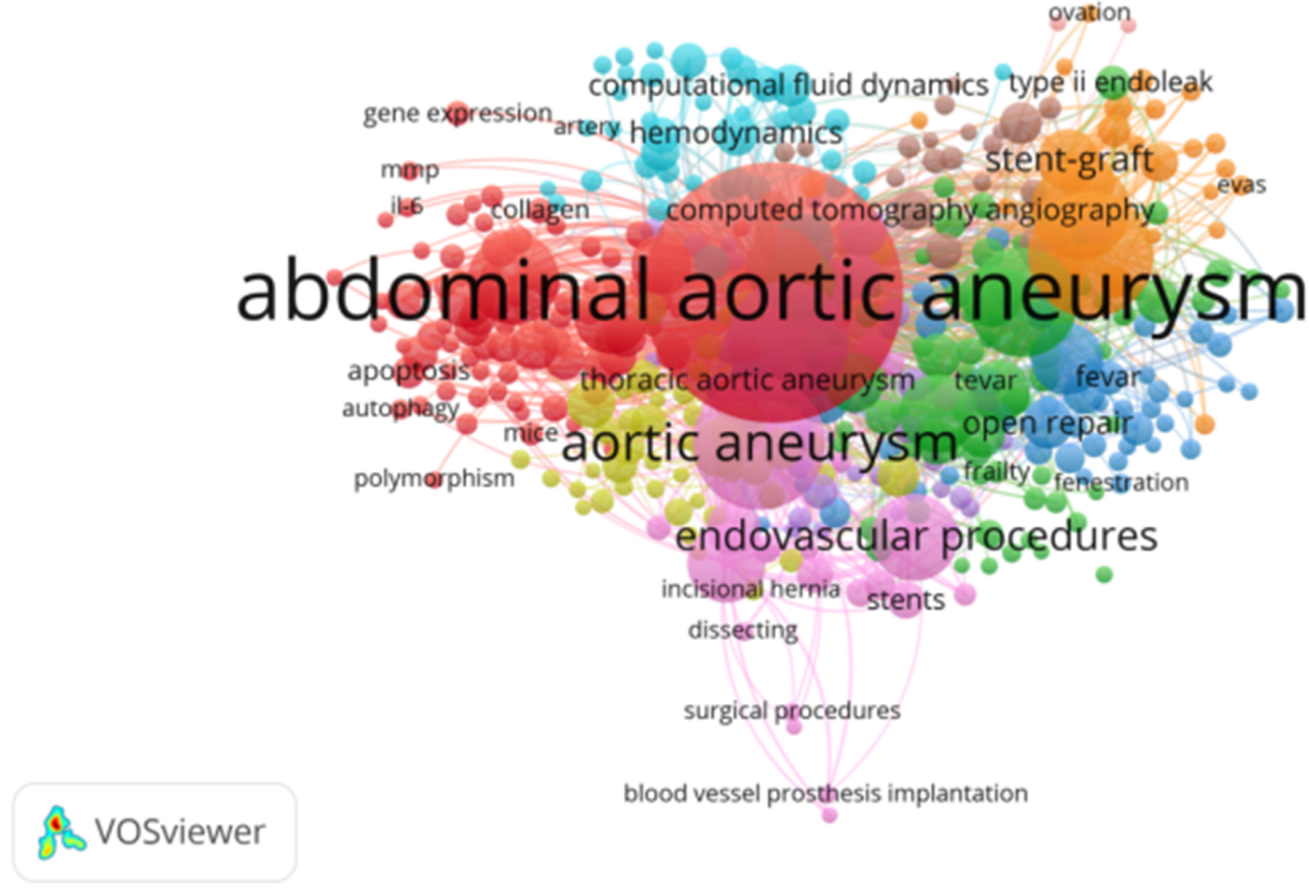
The co-occurrence analysis of author keywords.

Key words reflect the concept of the topic of the paper, which can reflect the research hotspots and core content of the studied field. According to the co-occurrence analysis, there were 11,799 keywords related to abdominal aortic aneurysm, with the top 10 keywords being abdominal aortic aneurysm, aortic aneurysm, endovascular aneurysm repair, aneurysm, EVAR, endoleak, inflammation, endovascular procedures, abdominal, and mortality (Table 3 and Figure 6).

**Table 3:** The author keywords in the studies of the abdominal aortic aneurysm.

Rank	Keywords	Counts
1	Abdominal Aortic Aneurysm	2,492
2	Aortic Aneunysm	656
3	Endovascular Aneurysm Repair	585
4	Aneurysm	558
5	EVAR	449
6	Endoleak	366
7	Inflammation	313
8	Endovascular Procedures	280
9	Abdominal	231
10	Mortality	227

### 3.4 Analysis of cited references, cited sources, and cited authors

**Figure 7:**
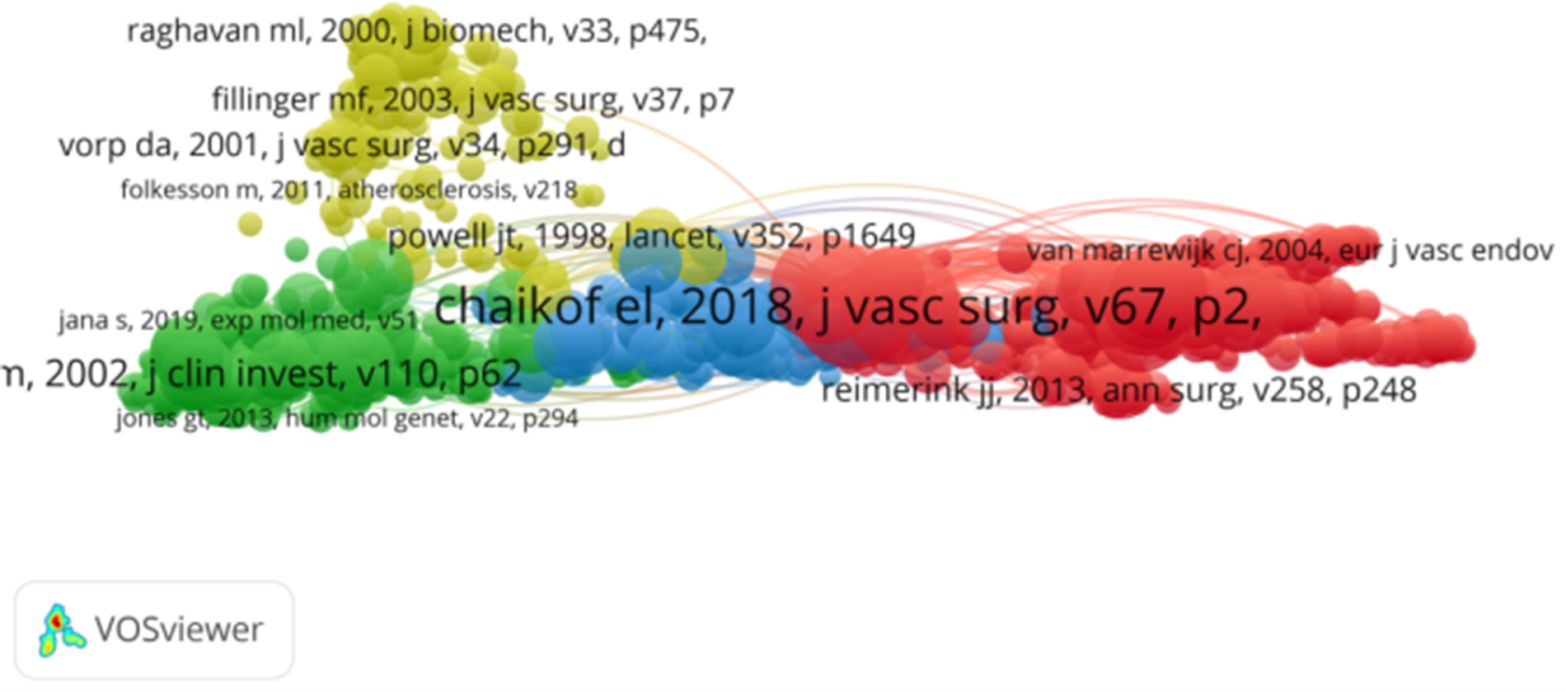
The visualization ofco-cited references on research of abdominal aortic aneurysm.

**Figure 8:**
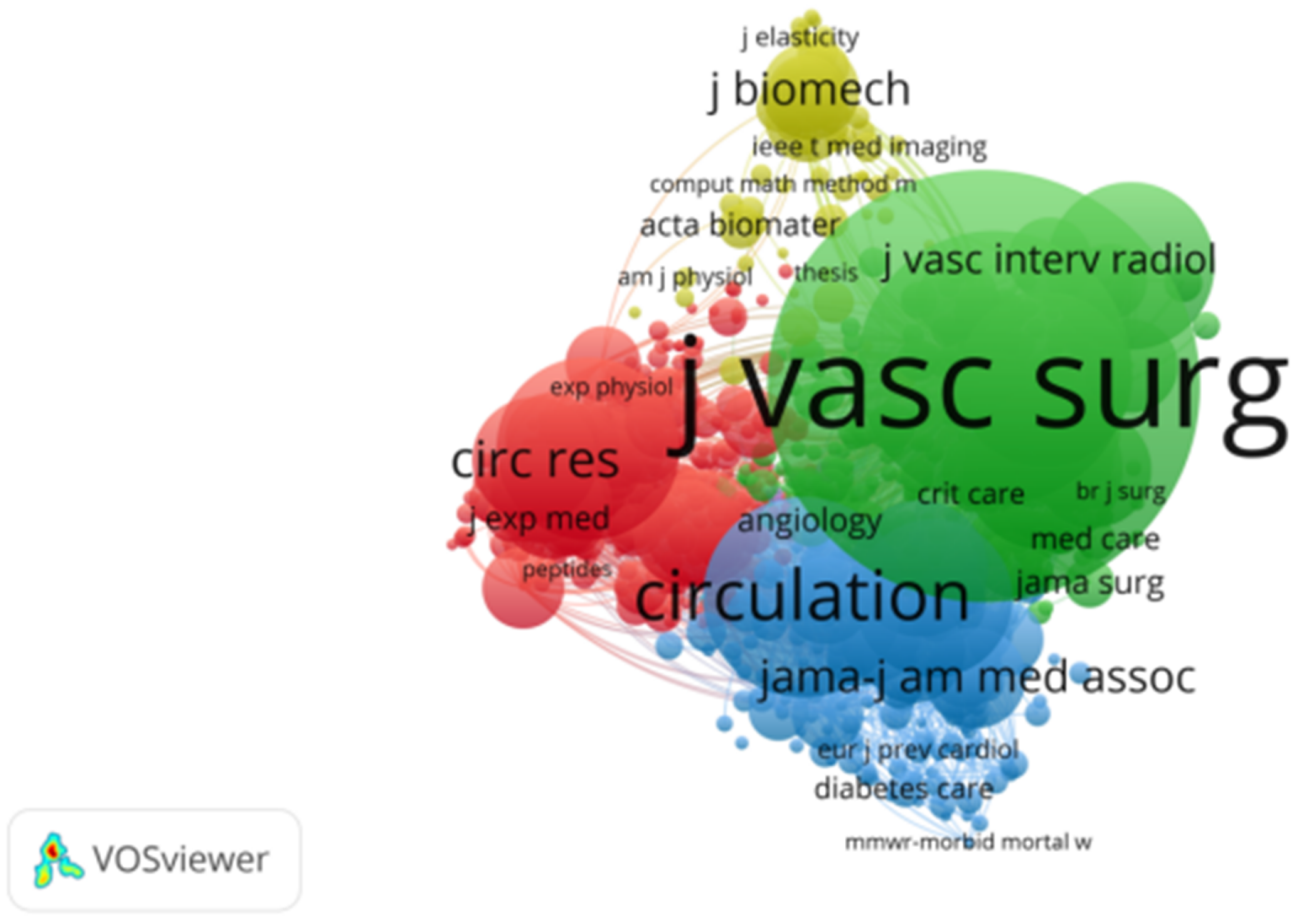
The visualization ofco-cited journals on research ofabdominal aortic aneurysm.

**Figure 9:**
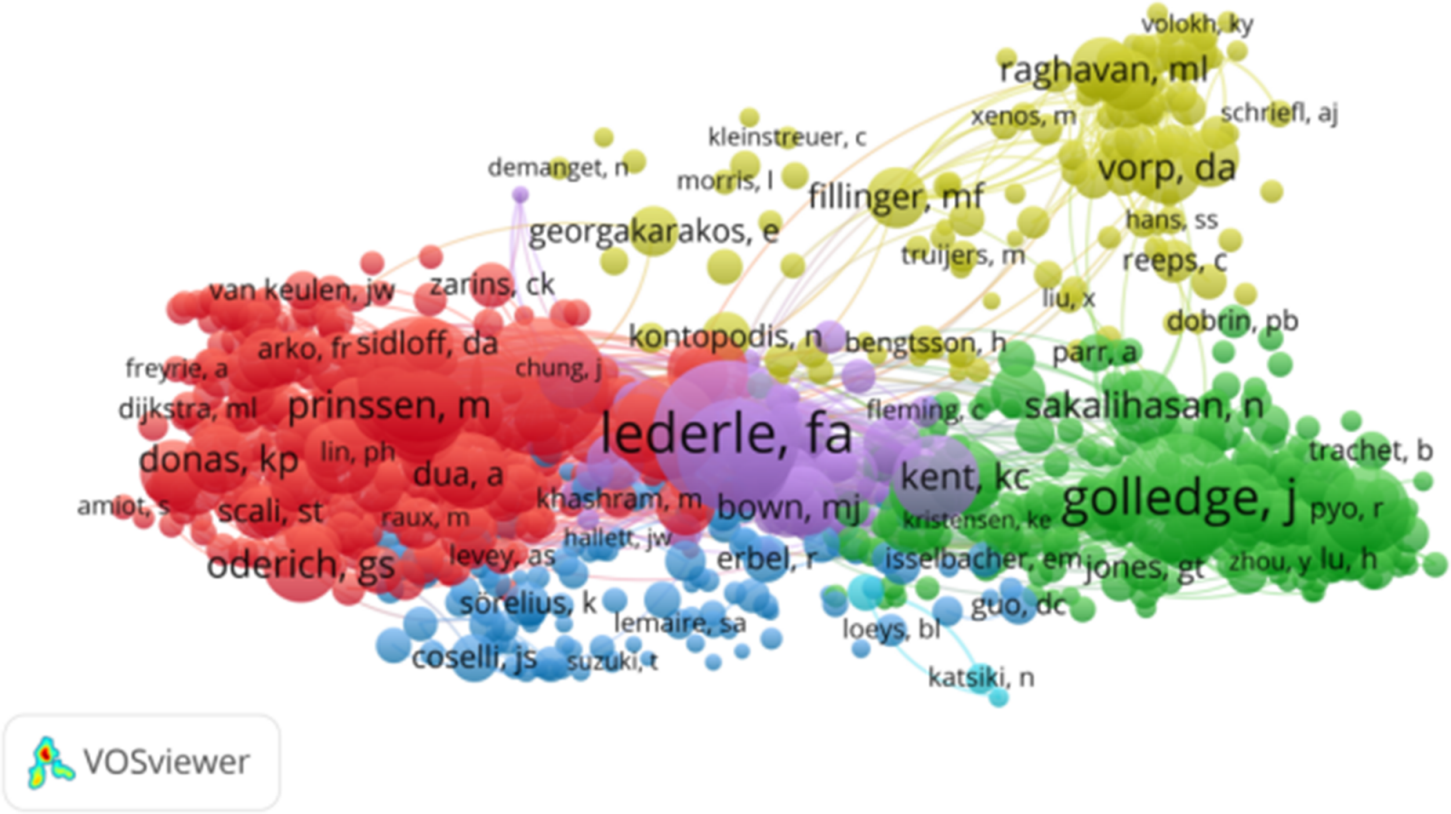
The visualization of co-cited authors on research of abdominal aortic aneurysm.

From 1 January 2014, to 1 March 2024, a total of 178,821 studies on abdominal aortic aneurysm were cited. Among the top 10 cited references, all were cited at least 400 times. “Chaikof et al, J Vasc Surg. 2018 Jan; 67(1):2–77.e2.”, “Wanhainen A, Eur J Vasc Endovasc Surg. 2019 Jan; 57(1):8–93.” and “Moll FL, Eur J Vasc Endovasc Surg. 2011 Jan; 41 Suppl 1:S1-S58.” The constructed network of cocited references is shown in Figure 7. Of the 17,762 cited journals, the top ten were “J Vasc Surg” (52,708), “Eur J Vasc Endovasc” (21,215), “Circulation” (11,427), “Arterioscl Throm Vas” (9,522), and “Ann Vasc Surg” (8,661). “J Endovasc Ther” (7,961), “New Engl J Med” (7,142), “Brit J Surg” (7,087), “Lancet” (5,264), and “Circ Res” (4,467), as shown in Table 4. The network of cocited journals is shown in Figure 8. Among the 108,565 authors cited, the top 10 were cited at least 700 times. The most common cocitation was Lederle, FA (2,435), followed by Chaikof, EL (2,143), Golledge, J (1,848), Greenhalgh, RM (1,754), and Wanhainen, A (1,443). A network of cocited authors was constructed (Figure 9) and clearly shows that there is also active cooperation among different cocited authors.

**Table 4:** The top 10 cited-journals related to abdominal aortic aneurysm.

Rank	cited-journal	Counts
1	j vasc surg	52,708
2	eur j vasc endovasc	21,215
3	circulation	11,427
4	arterioscl throm vas	9,522
5	ann vasc surg	8,661
6	j endovasc ther	7,961
7	new engl j med	7,142
8	brit j surg	7,087
9	lancet	5,264
10	circ res	4,467

### 3.5 Trend topics

**Figure 10:**
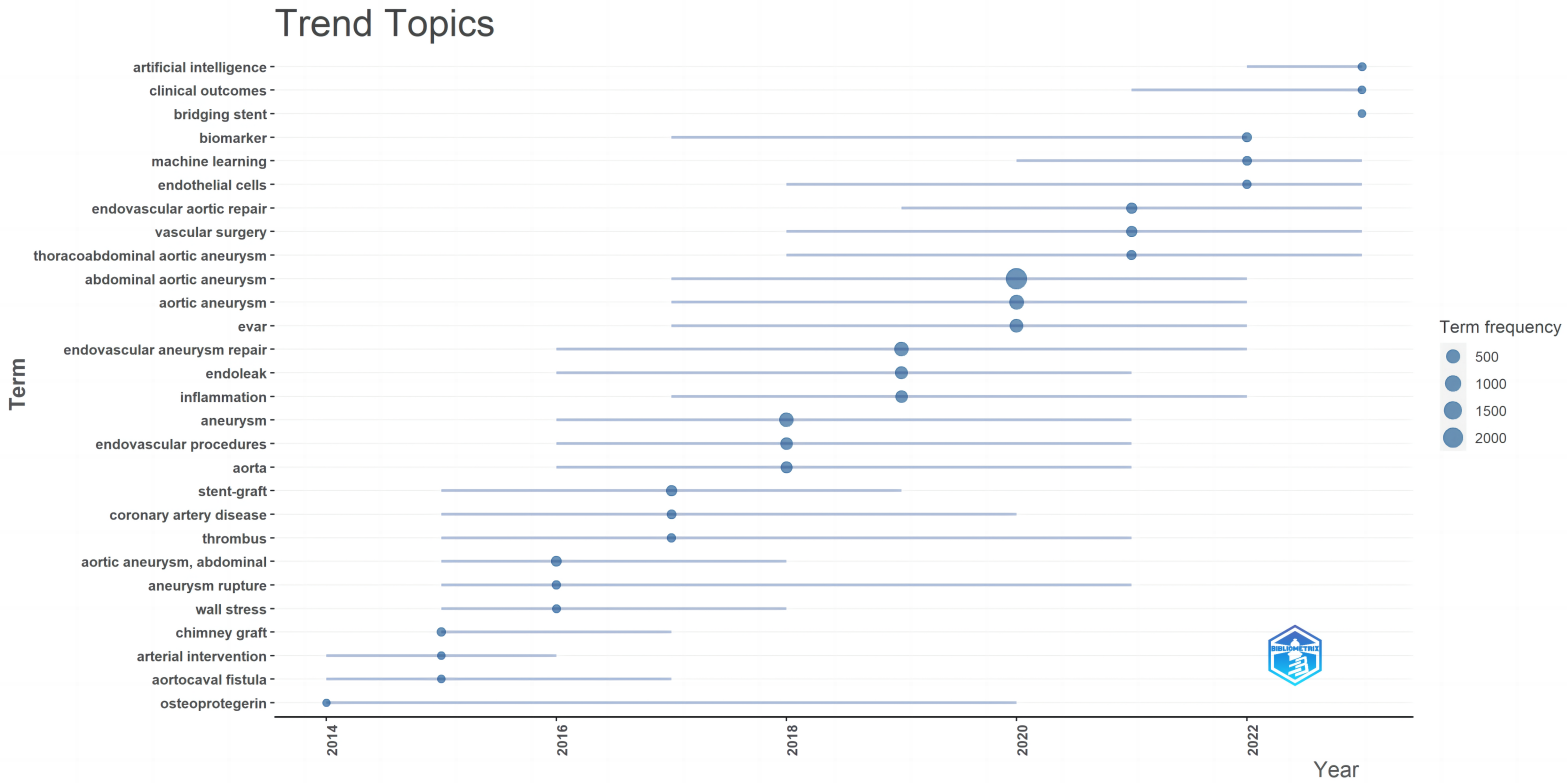
Trend topic analysis.

Keyword trend thematic analysis (Figure 10) revealed that from 2014 to 2016, keywords mainly focused on “chimney graft”, “arterial intervention”, and “aortocaval fistula”. From 2016 to 2018, the major keywords were “stent-graft”, “coronary artery disease”, and “thrombus”. The main keywords from 2018 to 2020 were “endovascular aneurysm repair”, “endoleak”, and “inflammation”. The most common keywords from 2020 to 2022 were “vascular surgery”, “thoracoabdominal aortic aneurysm”, and “abdominal aortic aneurysm”. we found that the earliest article about “bridging stent” was published in 2008, and the earliest article about “chimney graft” was also published in 2008. In 2011, a scholar found that minimally invasive treatment with the fast-track protocol may be a valid alternative to EVAR. At the same time, In 2017, some scholars compared patients undergoing fast-track abdominal aortic surgery (OPEN group) and endovascular aneurysm repair (EVAR group) and found that Minimally invasive treatment with the fast-track protocol and EVAR are both valid options in octogenarian patients because we obtained comparable results in terms of resumption of feeding, early ambulation, days of hospitalization, perioperative rate of mortality and morbidity. And we added it to the manuscript. At present, “artificial intelligence”, “clinical outcomes” and “bridging stent” are the three keywords with the highest frequency over the past two years, indicating that these three keywords may be the hotspots in recent research on abdominal aortic aneurysm.

### 3.6 Reference with citation bursts

**Figure 11:**
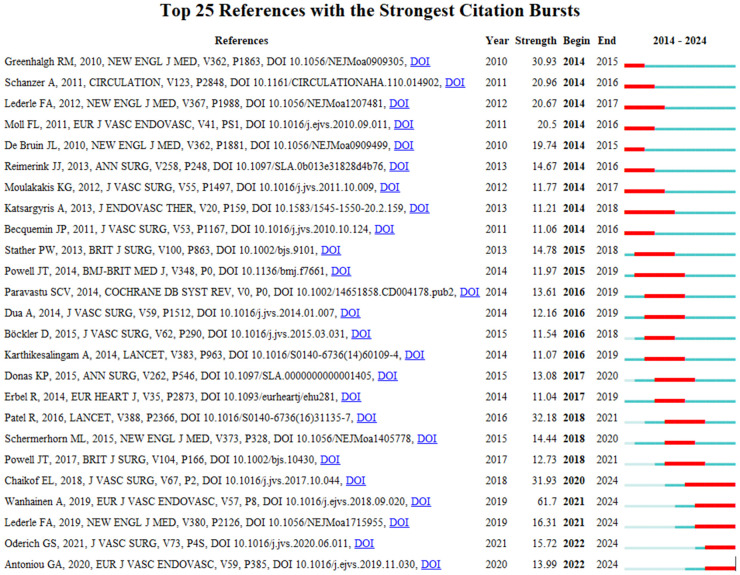
Top 25 references with strong citation bursts.

In our bibliometric study on abdominal aortic aneurysm, 25 highly cited studies were identified through CiteSpace. The citation bursts occurred as early as 2010 and as late as 2019. The title of the reference with the strongest citation burst (strength = 138) was “European Society for Vascular Surgery (ESVS) 2019 Clinical Practice Guidelines on the Management of Abdominal Aorto-iliac Artery Aneurysms”. The reference with the second strongest (strength = 138) citation burst was F L Moll et al., “Management of abdominal aortic aneurysms clinical practice guidelines of the European Society for Vascular Surgery”, and the reference with the third strongest citation burst was “Endovascular vs. open repair of abdominal aortic aneurysm” by Greenhalgh RM, et al. There were 25 references with burst power ranging from 30.72 to 138 (Figure 11).

## 4 Discussion

### 4.1 General information

Through bibliometric analysis, we can quickly understand the current research status of a disease, including research hotspots and the latest research direction. To date, no comprehensive bibliometric study of abdominal aortic aneurysms has been conducted. We used relevant bibliometric research methods and software to analyze and visualize the literature on abdominal aortic aneurysms published from 1 January 2014, to 1 March 2024, aiming to understand the latest research status and direction, provide ideas for future research, and guide treatment.

In this study, VOSviewer, the R package and CiteSpace software were used to conduct bibliometric analysis of the trends and hotspots of abdominal aortic aneurysm research. We retrieved 9,905 original articles and reviews by searching the Web of Science core collection database. According to statistics, the United States, China, the United Kingdom, Germany and Italy are the main contributors in this field. Among them, the United States has the highest number of publications, followed by China. With improvements in economic capacity and people's ability to understand the disease, as well as the update and reform of medical technology, treatment methods and innovations for abdominal aortic aneurysm have greatly improved, which has further increased the emergence of new research on this disease. China and the United States are incomparable countries, having populations and economic capacities among the largest in the world. Early and comprehensive research on the incidence and treatment of abdominal aortic aneurysm will greatly reduce the harm caused by this disease. At the same time, close cooperation and communication between countries with a large number of publications in this field will also be beneficial for the treatment of this disease. For high-yield countries, paper quality should also be considered; for future publications, quality and impact should be improved. Figure 4 shows that the United States, China, the United Kingdom, Japan and other countries have very close cooperative relationships, and many papers have been published on the basis of these relationships. Therefore, each of these countries have strengthened academic exchanges and cooperation to promote research on abdominal aortic aneurysm and development of treatments.

In terms of institutions, Stanford University, Harvard Medical School, and the Mayo Clinic have made great contributions to abdominal aortic aneurysm research, with Stanford University having the highest number of research results and important cooperative relationships with other institutions. Interestingly, the cocited author analysis showed that the most cocited author was “Lederle, FA”, a scholar from the Veterans Affairs Cooperative Study Group. At the same time, among the top ten institutions, there are no Chinese institutions on the list. In terms of the number of publications from China, the largest institution is China Medical University (86), ranking 18th, which indicates that in research on abdominal aortic aneurysm, Chinese scholars and their respective institutions should strengthen cooperation and exchange and strive to publish more research on abdominal aortic aneurysm.

The analysis of journal publication volume and cocited journals can provide researchers with appropriate submission plans and accelerate the publication of research results (22). The most cocited journal was J Vasc Surg (impact factor), which has considerable recognition and authority. In this study, the matching rate of the top five most productive journals and cocited journals was 60%, indicating that the quantity and quality of studies on abdominal aortic aneurysm are not uniform, further indicating that it is necessary to strengthen the cooperation between scholars and institutions to publish higher-quality articles and provide a reliable basis for the study of abdominal aortic aneurysm.

### 4.2 Knowledge base

Among the top 5 cited articles, 3 were related to clinical guidelines for the treatment and care of abdominal aortic aneurysm (23–25), and 2 were about the comparison between endovascular repair and open repair of abdominal aortic aneurysm (26, 27). The most cited guideline was “The Society for Vascular Surgery practice guidelines on the care of patients with an abdominal aortic aneurysm” published in the Journal of Vascular Surgery in 2018 (23). Interestingly, the most commonly cited guideline was not the treatment guideline but the nursing practice guideline for abdominal aortic aneurysm. This finding indicates that the treatment of abdominal aortic aneurysm is inextricably linked to patient care.

### 4.3 Hotspots and trends

Keyword co-occurrence can indicate hot spots in a certain field. Table 3 and Figure 6 show the common keywords related to abdominal aortic aneurysm. These keywords include abdominal aortic aneurysm, aortic aneurysm, endovascular aneurysm repair, aneurysm, EVAR, endoleak, inflammation, endovascular procedures, abdominal and mortality. These keywords may indicate potential research hotspots for abdominal aortic aneurysm treatment. According to the trend analysis, future research on abdominal aortic aneurysm may focus on the following aspects:

#### 4.3.1 Artificial intelligence

Abdominal aortic aneurysm is a serious life-threatening cardiovascular emergency, and surgical treatment is the only way to save patients' lives. At present, artificial intelligence is used in many fields (28–31). The application of artificial intelligence technology in the medical field can help researchers and clinicians further prevent, identify and treat diseases. Diseases involving abdominal aortic aneurysm originate from the large blood vessels of the human body, and CTA (32) and other imaging examinations are needed to identify and treat the disease. Because the shape and distortion of blood vessels are often not accurately measured before surgery, artificial intelligence may have an advantage. AI can be used to quantitatively calculate the formation, shape and distortion of blood vessels in patients with abdominal aortic aneurysm. It can even predict the risk of abdominal aortic aneurysm rupture (28, 33, 34). Moreover, artificial intelligence can be used to evaluate surgical indications and help surgeons make more beneficial treatment plans for patients. Therefore, in the era of big data where artificial intelligence is very popular, the combination of research on abdominal aortic aneurysm and artificial intelligence will be a promising research field, benefiting abdominal aortic aneurysm patients and opening up a new path for clinicians.

#### 4.3.2 Clinical outcomes

Clinical outcomes and long-term prognosis have always been the main criteria for evaluating the treatment of any disease, and the same is true for the treatment of abdominal aortic aneurysm (35–37). At present, there are two common surgical treatment methods for abdominal aortic aneurysm (AAA), endovascular aneurysm repair (EVAR) and artificial vessel replacement (AVR). According to relevant studies, the clinical prognoses and complications of EVAR and AVR exhibit some differences, including among different populations (38) and between males and females (39). At the same time, with the great improvement of the current stent types and the technical level of surgeons, both the treatment concept for abdominal aortic aneurysm and clinical outcomes have improved. Therefore, clinical outcomes of patients with abdominal aortic aneurysm will improve on the basis of technical updates and improvements in surgical skills. Knowledge of clinical outcomes of abdominal aortic aneurysm also needs to be updated; thus, clinical outcome is a good topic of future research on abdominal aortic aneurysm. We found that smoking cessation, well-controlled blood pressure, and good lifestyle habits were beneficial for the survival of patients with smaller abdominal aortic aneurysms. For surgical patients, weight loss, appropriate preoperative surgical methods, preoperative management of cardiovascular risk factors, and the use of statins can improve the long-term survival rate of abdominal aortic aneurysm surgery.

#### 4.3.3 Bridging stent

At present, the relationship between medicine and engineering is increasingly close, and for diseases such as abdominal aortic aneurysm that require endovascular operation, compliance with artificial blood vessels or stents is a key point in their treatment, and better compliance can effectively reduce the postoperative complications of such diseases (40, 41). In recent years, with the accelerated development of science and technology, the material, quality, and type of stents have been greatly improved, which not only improves ideas for medical researchers but also creates an opportunity for researchers who produce stents.

#### 4.3.4 Biomarkers

The pathological mechanisms underlying the occurrence and development of abdominal aortic aneurysm, including the inflammatory response, elastic fiber loss and collagen degradation, have been studied by many scholars (42, 43). Many biomarkers can be used for disease diagnosis and prognosis. At present, in terms of biomarkers, there are studies on the pathogenesis, prevention, drug treatment, surgical treatment and prognosis of abdominal aortic aneurysm (44, 45). It is clear that the study of biomarkers for abdominal aortic aneurysm has been beneficial, providing basic medical scientists with research ideas.

### 4.4 Limitations

This study is the first to systematically analyze and summarize the current hot topics related to abdominal aortic aneurysm via a bibliometric approach, which provides some ideas for scholars who study abdominal aortic aneurysm-related research. We used VOSviewer, the R package and CiteSpace for in-depth analysis, and the research results were valid and reliable. However, this study also has certain limitations. First, we only searched the Web of Science core collection database and did not search other databases (46, 47). Second, we included only articles published in English, which to some extent ignored meaningful literature in other languages.

## 5 Conclusion

This study is the first to systematically analyze and summarize acute abdominal aortic aneurysm using bibliometric methods. In recent years, with the increasing number of treatment methods for abdominal aortic aneurysm, research on abdominal aortic aneurysms has increased, and the number of articles published throughout the years has stabilized at approximately 1,000, indicating that research on abdominal aortic aneurysm has become increasingly prominent. China and the United States are still the main contributors to abdominal aortic aneurysm research, and more attention to cooperative efforts would further improve the value of abdominal aortic aneurysm research. In addition, in future research, researchers should strengthen national, institutional and multicenter cooperation, which can not only sublimate the technology and increase study sample sizes but also lead to the publication of more and higher quality articles. Further cooperation with artificial intelligence, as well as attention to the role of biomarkers in the occurrence, development, identification and prognosis of abdominal aortic aneurysm, will further improve the disease model of abdominal aortic aneurysm, providing broader, wider and more meaningful ideas for exploration of the treatment of abdominal aortic aneurysm.

## Data Availability

The original contributions presented in the study are included in the article/Supplementary Material, further inquiries can be directed to the corresponding authors.
